# *N*-Acetyl-Aspartate Level is Decreased in the Prefrontal Cortex in Subjects At-Risk for Schizophrenia

**DOI:** 10.3389/fpsyt.2013.00099

**Published:** 2013-09-05

**Authors:** Marine Mondino, Jerome Brunelin, Mohamed Saoud

**Affiliations:** ^1^EA4615, CH le Vinatier, Université Claude Bernard Lyon 1, Lyon, France

**Keywords:** schizophrenia, vulnerability, risk, NAA, MRS, prefrontal cortex

## Abstract

Reduced *N*-acetyl-aspartate (NAA) levels have been reported in the prefrontal cortex (PFC) in patients with schizophrenia using proton magnetic resonance spectroscopy. However, it is unclear whether this NAA reduction predates the illness onset and is reported in subjects at-risk for developing schizophrenia (HRS). The aim of this study was to assess NAA levels in the PFC in HRS. We hypothesized that HRS display lower NAA levels than healthy controls in the PFC. Studies assessing levels of NAA/Creatine (NAA/Cr) in the PFC in HRS were extracted from literature. Meta-analysis tools were used to compute effect sizes of nine selected studies meeting our inclusion criteria (clinical and/or genetic HRS, groups of HRS, and healthy controls matched for age and gender, spectral acquisition in the PFC). We reported that HRS exhibited a significant lower NAA/Cr level (2.15 ± 0.29; *n* = 208) than healthy controls (2.21 ± 0.32; *n* = 234) in the PFC with a medium pooled effect size [Hedges’s *g* = −0.42; 95% confidence interval: (−0.61; −0.23); *p* < 0.0001] corresponding to an average 5.7% of NAA/Cr decrease. Secondary analysis revealed that this reduction was observed in young HRS (<40 years old) who have not reached the peak age of risk for schizophrenia (−11%, *g* = −0.82, *p* < 0.00001) but not in old HRS (>40 years old) who have already passed the peak age (*g* = 0.11, *p* = 0.56), when they are compared with their matched healthy controls. Our findings suggest that the NAA/Cr reduction in the PFC reported in patients with schizophrenia is observable only in HRS who have not passed the peak age of risk for schizophrenia. NAA/Cr level in the PFC could therefore be considered as a biological vulnerability marker of schizophrenia.

## Introduction

The prefrontal cortex (PFC) is crucially involved in schizophrenia (1). Morphological and functional abnormalities have been reported in the PFC at different stages of the illness (2, 3). Neurochemical substrates of these abnormalities can be investigated *in vivo* by assessing brain metabolite concentrations with proton Magnetic Resonance Spectroscopy (^1^H-MRS). Amongst the brain metabolites measured with ^1^H-MRS, the *N*-acetyl-aspartate (NAA) is a metabolite reflecting neuronal health or viability (4). NAA is the second most abundant metabolite in the Human brain and emits the strongest signal in ^1^H-MRS. NAA is detected in the adult brain only in neurons. Its precise functions are still under investigation, ranging from osmolyte to fluid balance in the brain, source of acetate for myelin synthesis, contributor to energy production in mitochondria, and precursor for *N*-Acetylaspartylglutamic acid (NAAG) synthesis. NAA decreases have been detected in several human disorders involving neuronal loss or dysfunction such as Alzheimer disease, epilepsy, amyotrophic lateral sclerosis, multiple sclerosis, AIDS, traumatic brain injury, Rett syndrome, mood disorder, stroke, and non-neuronal brain tumors such as glioma. In a recent review and metaanalysis of 64 studies including 1256 patients and 1209 healthy controls, Steen and colleagues (5) have reported a 10% NAA reduction in the PFC in patients with schizophrenia, compared with healthy controls. Such a NAA reduction may reflect neuronal or axonal loss and may account for neuronal and molecular perturbations leading to abnormal brain activity often reported in patients with schizophrenia (6). Reduced NAA levels in the PFC have also been observed in patients during acute stages of the illness (7) and in stabilized patients (8). Moreover, reduced NAA levels in the PFC have been associated with poor prognosis across the first years of the illness (9). Antipsychotic medication have been reported to modulate NAA concentration leading to the hypothesis that the changes observed in NAA levels in the PFC in patients may be due primarily to treatment (10).

According to the neurodevelopmental model of schizophrenia (11), some abnormalities predate the illness onset by several years. These abnormalities, considered as vulnerability markers of schizophrenia, are observed in ill, stabilized, and recovered patients with schizophrenia, as well as in individuals at high risk for schizophrenia [HRS; (12)]. HRS are either defined as clinical high-risk subjects or genetic high-risk subjects and are generally young adults or teenagers. Clinical high-risk subjects are people who developed a brief psychotic episode (<7 days) resolved without any intervention or people who exhibited schizotypal traits, i.e., subthreshold non-clinical psychotic symptoms. Genetic high-risk subjects are first or second-degree relatives of patients with schizophrenia, more frequently unaffected siblings of patients (13). Finally, HRS can also present the two types of risk: a genetic risk for psychosis coupled with deterioration in global functioning over the past year (Genetic Risk and Deterioration Syndrome). In HRS, the risk to develop the illness reaches a peak between 15 and 30 years old (it is admitted that the peak age of risk is reached at age 25) and then decreases with aging until 40 (14). After 40, the risk to develop the illness is considered as low and already passed. HRS displayed behavioral, cognitive, and brain abnormalities when they are compared with healthy subjects (15). Abnormalities observed in HRS usually have an intermediate magnitude between the frank deficit reported in patients with schizophrenia and “normal” functioning observed in healthy subjects. For instance, unaffected siblings of patients displayed cognitive abnormalities similar to those seen in probands but at lower intensity [e.g. (13, 16)]. Depending on the stability of their magnitude across the different stages of the illness, these abnormalities can be considered as trait, state, or intermediate vulnerability markers (16). Some morphological (17) and functional (18) brain abnormalities have been reported in the PFC in HRS. However, chemical substrates of such abnormalities remains unclear and ^1^H-MRS studies investigating NAA levels in the PFC in HRS led to inconsistent results. The aim of the current study was to investigate NAA levels in the PFC in HRS. To achieve this goal, we reviewed ^1^H-MRS studies assessing NAA levels in the PFC in HRS compared with healthy controls. We hypothesized that HRS displayed lower NAA levels in the PFC than healthy controls, suggesting an alteration in neuronal integrity in the PFC before the onset of frank psychotic episode and long-term medication intake. Since aging influences the risk to develop schizophrenia (13, 14), we hypothesized that NAA levels were especially lower in younger HRS who have not passed the peak age of risk for schizophrenia.

## Materials and Methods

### Selection of studies and exclusion criteria

A systematic search of the literature was conducted using Pubmed and Sciencedirect databases until March 2013. The article identification was based on various combinations of the following keywords: (“NAA” OR “*N*-acetylaspartate” OR “*N*-Acetylaspartic acid”) AND (“at-risk” OR “vulnerability” OR “liability” OR “ARMS”) AND (“schizophrenia” OR “psychosis” OR “schizotypy”) AND (“spectroscopy” OR “MRS”). The search was limited to original English-written articles related to research in human subjects. This primary search yielded 11 articles and was completed by a manual search from references cited in primarily found articles. This secondary search yielded 20 articles.

^1^H-MRS studies require selection of a voxel for spectrum acquisition. Many studies in HRS acquire spectra from different brain regions or subregions and with different voxel sizes, leading to difficulties in comparing findings. In order to tabulate NAA levels in a way that would enable us to compare between studies, only studies expressing or allowing to compute NAA concentrations as a ratio to creatine (Cr) were included. Cr represents components of the cell’s energy metabolism and is assumed to remain relatively constant. Moreover, only studies investigating at least HRS and healthy control groups matched for gender and age were included. Among the selected articles, nine studies were excluded for not reporting original data in HRS (*n* = 6) and/or for not reporting NAA/Cr in our region of interest, the PFC (*n* = 3). Two studies were excluded because the group of HRS and the group of healthy controls were not matched for age.

A final total of nine articles were retained for analysis. Characteristics of studies are described in Table 1 (participants demographical characteristics and volume of the voxel placed in the PFC for ^1^H-MRS acquisition). The study of Jessen and colleagues (19) included two different groups of HRS according to the German Research Network on Schizophrenia criteria (20): early at-risk subjects (ER) and late at-risk subjects (LR). These two groups of subjects were considered separately in our analysis.

**Table 1:** **Characteristics of studies included in the analysis**.

	HRS			Healthy controls			Voxel (cm^3^)
	Age (years)	SD age	M/F	Age (years)	SD age	M/F	
Keshavan et al. (21)	15.1	2.7	5/4	14.3	5.4	6/4	3
Callicott et al. (22)	34.6	8.6	25/35	32.9	8.2	42/24	PFC
Brooks et al. (23)	11.0	1.7	9/7	10.8	1.7	6/6	8
Block et al. (24)	49.2	15.4	19/16	40.2	15.3	7/12	30
Tibbo et al. (25)	16.4	2.0	7/13	16.7	1.7	9/13	2.5
Jessen et al. (19) (ER)	27.0	6.8	5/5	34.8	13.5	17/7	30
Jessen et al. (19) (LR)	28.7	7.0	4/5	34.8	13.5	17/7	30
Purdon et al. (26)	46.3	6.1	2/13	43.5	6.75	3/11	15.6
Yoo et al. (27)	22.6	5.3	12/10	23.1	4.8	13/9	6
Lutkenhoff et al. (28)	49.5	10.0	7/5	55.7	3.8	6/15	8
Total	31.1	7.3	95/113	29.5	6.1	126/108	–

*Cr, creatine; ER, early risk; HRS, high-risk subjects; LR, late-risk; M/F, number of male/female; NAA, *N*-acetyl-aspartate; voxel, voxel size in cm^3^; SD, standard deviation*.

### Effect size calculation and data analysis

Comparisons between groups were realized using two-tailed paired Student’s *t*-test for age and *chi square Fischer’s exact* test for gender.

Effect sizes were computed as the standardized mean difference and standard deviation between HRS and their matched control groups in each study. An individual corrected effect size for each study was calculated (29), and a combined (pooled weighted) effect size was obtained through the implementation of a random effect model. According to Cohen (30), an effect size of 0.2 can be considered as small, 0.5 as medium, and 0.8 as large. Significance of effect sizes was assessed using two-tailed Student’s *t*-tests with a significance set at *p* < 0.05.

#### Effect of aging

Since aging influences the risk to develop schizophrenia [decrease of risk between 30 and 40; less risk after 41 years old; (13, 14)] as well as NAA levels [between before and after 40 years old; (31)], secondary analyses were conducted for studies reporting a mean age of HRS group older than 40 (who have already passed the peak age of risk for schizophrenia) and studies reporting a mean age of HRS group younger than 40.

## Results

The final sample was composed by 208 HRS (clinical and/or genetic HRS) and 234 healthy controls. Studies investigated NAA and Cr levels in the whole PFC (22) or in a voxel of variable dimensions placed in the PFC (21), the left PFC (19, 23, 24, 28), the left dorsolateral PFC (27), the right and left medial PFC (26), or the right medial PFC (25). Characteristics of studies were reported in Table 1.

No difference in age was reported between the HRS group (mean = 31.05 ± SD = 7.26) and the healthy control group (29.55 ± 6.07; Student’s *t*-test, *p* = 0.97). No difference in gender proportion was reported between the two groups (95 males and 113 females in the HRS group versus 126 males and 108 females in the healthy control groups, *Chi square test*, *p* = 0.10).

Seven of the selected studies did not show an individual significant difference between HRS and healthy controls [(19, 21, 24, 25) ER subgroup; (26, 27, 28)]. Three studies reported individual differences between groups [(19, 22, 23) LR subgroup]. Details are given in Table 2.

**Table 2:** **Effect size of studies investigating NAA/Cr in high-risk subjects for schizophrenia and healthy controls**.

	HRS			Healthy controls			Hedges’s *g*	95% CI		p*
	Mean	*n*	SD	Mean	*n*	SD		Lower	Upper	
Keshavan et al. (21)	1.1	9	0.27	1.14	10	0.24	−0.15	−1.05	0.75	0.74
Callicott et al. (22)	2.66	60	0.38	2.81	66	0.37	−0.40	−0.75	−0.05	0.03*
Brooks et al. (23)	1.67	16	0.22	1.92	12	0.31	−0.93	−1.71	−0.14	0.02*
Block et al. (24)	2.76	35	0.37	2.93	19	0.35	−0.46	−1.03	0.10	0.11
Tibbo et al. (25)	1.74	20	0.20	1.67	22	0.20	0.34	−0.27	0.95	0.26
Jessen et al. (19) (ER)	2.8	10	0.27	3.01	24	0.34	−0.64	−1.39	0.12	0.09
Jessen et al. (19) (LR)	2.54	9	0.33	3.01	24	0.34	−1.36	−2.19	−0.53	0.001*
Purdon et al. (26)	1.67	15	0.24	1.57	14	0.13	0.50	−0.24	1.24	0.18
Yoo et al. (27)	1.3	22	0.23	1.37	22	0.13	−0.37	−0.96	0.23	0.22
Lutkenhoff et al. (28)	1.47	12	0.24	1.29	21	0.32	0.60	−0.13	1.32	0.10
Total	2.15	208	0.29	2.28	234	0.32	−0.42	−0.61	−0.23	0.0001*

*Significant difference, two-tailed Student’s *t*-test.

*CI, confidence interval; ER, early risk; HRS, high-risk subjects; LR, late-risk; *n*, sample size; SD, standard deviation*.

**Figure 1:**
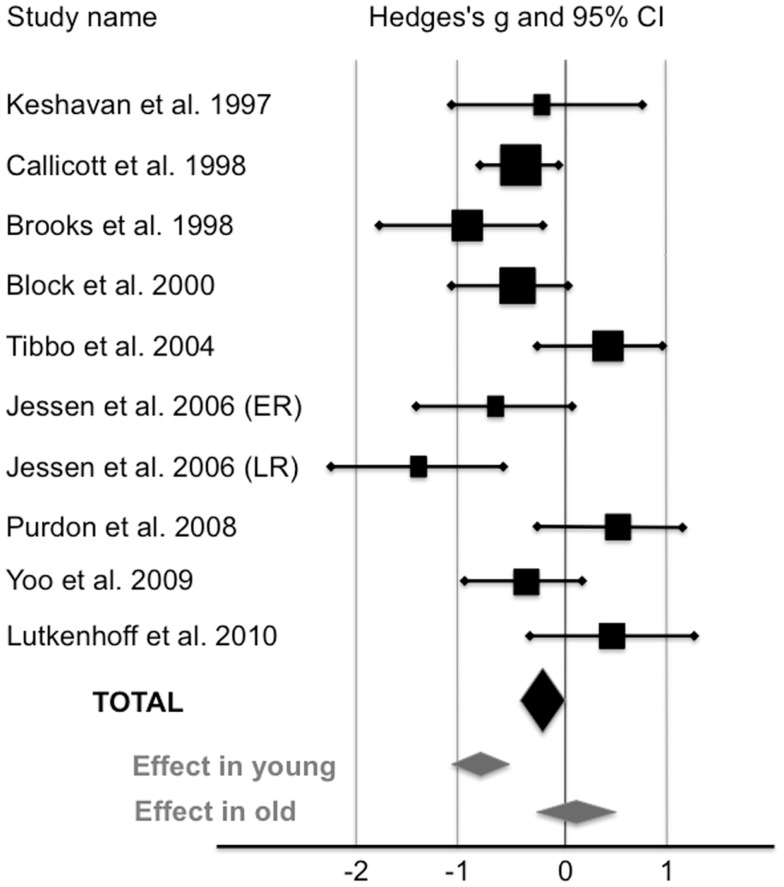
**Levels of NAA/Cr in the prefrontal cortex of high-risk subjects compared with healthy controls (random-effects model)**. The horizontal line represents the confidence interval for each study, and each square represents the point estimate. The size of the square corresponds to the weight of the study. The diamond represents the pooled effect size estimate in the whole HRS sample (black diamond), in young HRS (i.e., who have not reached the peak age of risk for schizophrenia;<40), and in old HRS (i.e., who have already passed the peak age of risk for schizophrenia;>40) (gray diamonds).

Compared with healthy controls, HRS displayed a significant 5.7% reduction of NAA/Cr in the PFC (see Figure 1; Table 2). The mean NAA/Cr was significantly lower in the HRS group (2.15 ± 0.29) than in the healthy controls group (2.28 ± 0.32). The random effect model showed a significant medium pooled effect size of −0.42 [95% confidence interval (CI): (−0.61; −0.23); *p* < 0.0001].

### Effect of aging

Three studies reported a mean age of subjects older than 40 (24, 26, 28). When these studies were removed, HRS exhibited a significant 11% reduction of NAA/Cr in the PFC. NAA/Cr was lower in younger HRS (2.12 ± 0.29; *n* = 146) than in younger controls (2.38 ± 0.32; *n* = 180) with a large pooled effect size of −0.82 [95% CI: (−1.08; −0.62); *p* < 0.000001]. In studies reported a mean age of subjects older than 40, NAA/Cr was not different between HRS (1.96 ± 0.28; *n* = 62) and healthy controls (1.93 ± 0.27; *n* = 54) with an effect size of 0.11 [95% CI: (−0.26; 0.47); *p* = 0.56].

## Discussion

We conducted an analysis on 9 studies assessing NAA/Cr level in the PFC in HRS (10 groups) and in matched healthy controls. We reported a significant 5.7% reduction of NAA/Cr in HRS compared with healthy controls. The effect size of this reduction was medium (*g* = −0.42). Secondary analyses revealed a large effect size between HRS who have not reached the peak age of risk for schizophrenia (*g* = −0.82) and their matched healthy controls. Conversely, no difference was reported between HRS who have already passed the peak age of risk for schizophrenia (older than 40) and their matched healthy controls (*g* = 0.11). These results suggest that NAA/Cr in the PFC is reduced only in young HRS. Noteworthy, the NAA/Cr reduction reported in the PFC in young HRS (−11%) seems similar to the reduction reported in patients with schizophrenia [−10%; (5)]. These results did not corroborate results from the metaanalysis of Brugger et al. (32), which reported no difference in NAA/Cr level between HRS and healthy controls. We assume that discrepancies between results can be explained by the difference in age of participants. Indeed, the metaanalysis conducted by Brugger and colleagues took into account studies including HRS who have passed the peak of risk for developing schizophrenia (24, 26, 28) and studies including groups of HRS and healthy controls that were not matched for age (33, 34).

Several limitations have to be acknowledged in our study. First, the volume and placement of the voxel used for assessing NAA levels were widely different amongst the included studies, from the whole PFC (22) to a voxel of 2.5 cm^3^ (25). Although this approach may mask findings that are specific to a particular subregion of the PFC, it has been reported in patients with schizophrenia that NAA findings were similar within each subregion of the PFC (5). Second, the power of the magnetic field used to acquire ^1^H-MRS spectra differed across the studies [3T ^1^H-MRS scan: (25, 26, 28), versus 1.5T in the other studies]. However, this difference was addressed by expressing NAA as a ratio to Cr instead of raw concentrations (35, 36). As shown in Table 2, there are wide variations in NAA/Cr ratios among studies (from 1.1 to 3.01 in the healthy subjects group and from 1.1 to 2.80 in the HRS group). This discrepancy may have resulted from methodological differences in ^1^H-MRS acquisitions (e.g., voxel size, power of the magnetic field). However, such variations may not have impact the comparison between groups of HRS and healthy controls since values were normalized between matched groups (5). In the sample of HRS, some patients were treated with antipsychotic medication. It was not possible to split HRS into treated versus untreated based on information provided in the papers reviewed. Since antipsychotic are known to modulate NAA level in patients with schizophrenia (10), we cannot exclude an effect of medication on NAA level in our sample of participants. However, it seems that antipsychotic increased NAA level in patients with schizophrenia (37), thus the observed reduction in NAA/Cr in HRS cannot be explained by antipsychotic intake in HRS. A last limit consists in the heterogeneity of HRS samples included in the studies (e.g., offspring and siblings of patients with schizophrenia, subjects with prodromal or subthreshold clinical symptoms, subjects who will develop or have already developed other Axis I psychopathology, clinical high-risk subjects or subjects with a combination of different risk). Here again, it was not possible to split the sample in groups according to their level of risk based on information provided in the studies. This inherent limitation to our approach ecologically reflects the heterogeneity of HRS. Finally, even if the sample sizes of the included studies were rather limited, our final sample size can be considered as large enough to detect a 5.7% difference (208 HRS and 234 healthy control, statistical power 100%).

Hence, we reported that NAA/Cr in the PFC was lower in HRS than in healthy controls, especially in young HRS. This result suggests that the NAA/Cr reduction predates the onset of the illness and is not present in HRS who have already passed the peak age of risk for developing schizophrenia. This result is in line with studies reporting reduced prefrontal NAA/Cr in adolescents with first episode psychosis who later progress to schizophrenia but not in those who later progress to other disorders (38) or do not develop any psychiatric condition. The magnitude of the reduction was also proposed to be an early marker of outcome in first psychotic episode (9, 33). Thus, the NAA/Cr reduction in the PFC seems to be accurately detectable at each stage of the illness and to be specific to schizophrenia. Therefore it can be considered as a vulnerability marker of schizophrenia.

The NAA reduction may reflect a neuronal loss and/or dysfunction and may be linked to early neuronal damage, supporting the neurodevelopmental hypothesis of schizophrenia. However, some studies have reported an exacerbation of the NAA/Cr decrease in the PFC during the first years of the illness following the onset of psychosis (8, 39). The exacerbation of NAA/Cr reduction following the onset of the illness suggests an additional brain degenerative process superimposed on the developmental impairment (7, 40, 41).

## Conclusion

In sum, the NAA/Cr reduction observed in the PFC of HRS may be proposed as a biological marker of vulnerability to schizophrenia. Further longitudinal studies can help us determine if NAA/Cr in the PFC is able to predict transition to schizophrenia. Moreover, studies targeting other brain areas and/or other ^1^H-MRS measurable metabolites implicated in schizophrenia can propose patterns of metabolic alterations helpful to decipher physiological hypotheses.

## Conflict of Interest Statement

The authors declare that the research was conducted in the absence of any commercial or financial relationships that could be construed as a potential conflict of interest.
